# Post-Operative Delirium Masking Acute Angle Closure Glaucoma

**DOI:** 10.2478/jccm-2023-0016

**Published:** 2023-07-31

**Authors:** Zariel Jiaying Sim, Xing Jieyin, Thangavelautham Suhitharan

**Affiliations:** Singhealth, Singapore, Singapore; Singapore General Hospital, Singapore, Singapore

**Keywords:** acute angle closure glaucoma, delirium, general anaesthesia, post-operative, critical care

## Abstract

**Introduction:**

Acute angle closure glaucoma (AACG) is an ophthalmological emergency, and can lead to the devastating consequence of permanent vision loss if not detected and treated promptly. We present a case of an atypical presentation of unilateral AACG on post operative day (POD) 1, after a prolonged operation under general anaesthesia (GA).

**Case presentation:**

A 65-year-old female underwent a 16 hour long operation for breast cancer and developed an altered mental status with a left fixed dilated pupil on POD 1. She was intubated to secure her airway in view of a depressed consciousness level and admitted to the intensive care unit. Initial blood investigations and brain imaging were unremarkable. On subsequent review by the ophthalmologist, a raised intraocular pressure was noted and she was diagnosed with acute angle closure glaucoma. She was promptly started on intravenous acetazolamide and pressure-lowering ophthalmic drops. Her intraocular pressure normalized in the next 24 hours with improvement in her mental status to baseline.

**Conclusion:**

AACG needs to be consistently thought of as one of the top differentials in any post-operative patient with eye discomfort or abnormal ocular signs on examination. A referral to the ophthalmologist should be made promptly once AACG is suspected.

## Introduction

Acute angle closure glaucoma (AACG) is an ophthalmological emergency, and can lead to the devastating consequence of permanent vision loss if not detected and treated promptly. AACG occurs when there is a sudden pupillary block, preventing the outflow of aqueous humour and resulting in an increase in intraocular pressure. This typically presents with symptoms of sudden onset of severe ocular pain or headache, nausea and vomiting and a decrease in visual acuity [1]. We present a case of an atypical presentation of unilateral AACG on post-operative day (POD) 1, after a prolonged operation under general anaesthesia (GA).

## Case presentation

A 65-year-old female (ASA 3, past medical history of hypertension, hyperlipidaemia, right breast cancer, no known ophthalmological history) was admitted electively for right breast wall resection with reconstruction using rib plates, resection of sternocleidomastoid joint and medial clavicle, reconstruction with vascularized fibula flap and skin coverage with abdominal based flap. Her pre-operative blood investigations were unremarkable. She was induced with Propofol 100mg, Fentanyl 100mcg, Lignocaine 100mg and Atracurium 30mg. She was maintained on Desflurane, Remifentanil target-controlled-infusion (TCI), Morphine infusion and Atracurium infusion. She was transfused with 4 units of Packed Red Blood Cells. She was placed in a supine position throughout the 16-hour operation with regular turning of her head to prevent pressure sores. At the end of the operation, she was reversed with 1.25mg of neostigmine and 0.2mg of glycopyrrolate with smooth emergence. Post-operatively, she was transferred to the high dependency ward for closer monitoring. She was alert and comfortable on arrival to the ward. Routine blood investigations sent off post operatively showed a raised lactate of 4.0 mmol/L, raised total white blood cell count 21.20 × 10^9^/L with a neutrophilic shift of 87.5% (Table 1).

**Table 1.** Table with results of blood investigations

**Blood investigation**	**POD 0**	**11^th^ hour post-op**	**POD 1**
pH	7.34	-	-
pCO2 (mmHg)	39.5	-	-
pO2 (mmHg)	79.3	-	-
Bicarbonate (mmol/L)	20.7	-	-
Base Excess (mmol/L)	−4.5	-	-
Oxygen saturation (%)	94.5	-	-
Urea (mmol/L)	4.4	4.2	3.6
Sodium (mmol/L)	139	139	138
Potassium (mmol/L)	3.9	3.6	3.6
Chloride (mmol/L)	107	106	106
Creatinine (μmol/L)	45	33	32
Glucose (mmol/L)	12.9	10.9	9.3
Magnesium (mmol/L)	0.66	1.01	0.81
Calcium (mmol/L)	2.07	2.01	1.92
Phosphate inorganic (mmol/L)	1.12	0.78	0.69
Lactate (mmol/L)	4.0	2.3	1.0
Ketones (mmol/L)	0.0	-	-
Haemoglobin (g/dL)	13.3	13.3	-
White blood cell count (× 10^9^/L)	21.20	24.90	-
Neutrophil (%)	87.5	87.9	-
C-reactive protein (mg/L)	-	-	137
Procalcitonin (UG/L)	-	-	0.93

At the 11^th^ hour post-operatively, she was reviewed by the Plastic Surgery team. She appeared less responsive and was staring up at the ceiling. On examination, her temperature was 37.4 Celsius degrees, heart rate 120 beats per minute, blood pressure 188/87 mmHg, oxygen saturation of 100% on room air. Her Glasgow Coma Scale (GCS) was E4V3-4M6 and an examination of her pupils could not be done as she was actively closing her eyes. Blood investigations showed a raised White Blood Cell count of 24.9 × 10^9^/L and raised Lactate 2.3mmol/L (Table 1). Computed Tomography (CT) of the brain showed no acute intracranial haemorrhage, established territorial infarct or evidence of mass effect.

Overnight, she developed a temperature of 38.5 Celsius degrees. A blood test was repeated and showed a procalcitonin level of 0.93 UG/L and a C-reactive protein level of 137 mg/L (Table 1). Her GCS remained the same. Blood cultures were taken and she was started on IV cefazolin 2g. The following morning (POD 1), her GCS reduced to E4V1M4-5 and her left pupil appeared dilated. Intensive Care Unit (ICU) outreach team was called to review the patient.

On review, her GCS was E4V1M1. Her right pupil was 3mm reactive to light while her left pupil was 6mm unreactive to light. There was up rolling of her eyes but no limb jerking was noticed. In view of the depressed GCS of 6, she was intubated for airway protection. A CT imaging of the brain was repeated, and it was normal. She was admitted to ICU. An hour and a half after her admission to ICU, her GCS was E3VTM6 and she was moving her limbs symmetrically against gravity. Her right pupil was 2mm reactive to light and her left pupil remained dilated and non-reactive to light. She was reviewed by a neurologist who had concerns for a left sided surgical Cranial Nerve 3 palsy. An urgent Magnetic Resonance Imaging (MRI) of cranial nerves and Magnetic Resonance Angiography (MRA) performed showed no distinct lesion along the course of the ocular motor nerves and there was no aneurysm seen. On POD 1, she had instances of agitation and had attempted to remove drains and invasive lines. She was given haloperidol and restrained physically.

On POD 2, her consciousness level continued to fluctuate throughout the day between being able to make nonverbal communication with healthcare workers to not reacting to pain on supra-orbital pain stimulus. An electroencephalography conducted showed mild diffuse encephalopathy. In view of normal brain imaging findings, she was referred to the ophthalmologist for evaluation of possible ocular pathology as a cause for her dilated left pupil. Upon ophthalmology review on POD 2, her left eye globe was firm, with grade 1–2 Relative Afferent Pupillary Defect by reverse. The conjunctivae was injected with diffused corneal oedema and a hazy fundal view. The intraocular pressure (IOP) measured was above the upper limit (55 mmHg) of the Tono-Pen AVIA^®^ used. She was diagnosed with left primary acute angle closure glaucoma (AACG) and was started promptly on IV acetazolamide and pressure reducing ophthalmic drops - latanoprost, brimonidine tartrate, timolol, brinzolamide and prednisolone.

8 hours after the treatment for AACG was initiated, her IOP on the left was 31 mmHg and her left cornea appeared clearer. Her GCS improved to E4VTM6 and she was extubated. Overnight, she became disorientated and agitated, requiring IM haloperidol 5mg. The following morning (POD 3), her mental status improved and she was fully orientated. She was then stepped down to high dependency. On POD 5, her IOP normalised to 5 mmHg, visual acuity on the left however was 6/60 compared to 6/15 on the right. She underwent laser peripheral iridotomy. 2 weeks later, her vision improved to 6/9. She was continued on long term ophthalmic timolol. She recovered well and underwent rehabilitation.

## Discussion

There are several predisposing factors for glaucoma such as age greater than 60, female gender, East Asian ethnicity, and hyperopia [2]. Intra-operatively, IOP can increase due to prolonged prone positioning and drugs causing mydriasis. Anticholinergic medications such as atropine and glycopyrrolate, and sympathomimetic drugs such as phenylephrine and ephedrine have been associated with mydriasis [3]. The relevant risk factors our patient had were that of age, female gender and being of East Asian ethnicity. There was no previous ophthalmological examination before surgery and it is not routine for one to be done peri-operatively. None of the medications given post-operatively to our patient was associated with AACG. Hence, phenylephrine and glycopyrrolate used during her operation might have triggered her post-operative AACG. The detection of AACG in post-operative patients can be difficult as the use of analgesia in the peri-operative period can mask the headache and intraocular pain commonly felt. Patients might also be unable to communicate pain and visual symptoms due to sedation or altered mental status from post-operative delirium. In such circumstances, it would be helpful for clinicians to maintain a high degree of suspicion and rely on physiological signs of painful stimulus including that of hypertension and tachycardia, coupled with a thorough bedside ocular examination.

Peri-operative Neurocognitive Disorders (PND) is a spectrum of disorders, that includes impairment diagnosed pre-operatively, post-operative delirium, delayed neurocognitive disorder and post-operative neurocognitive disorder. Post-operative delirium occurs within the first week post operation [4]. Older age, presence of pre-operative cognitive impairment, major surgery [5], post-operative pain and discomfort [6] and the use of drugs such as benzodiazepines, opioids and antihistamine are risk factors for post-operative delirium [7]. Our patient's risk factors for post-operative delirium included her older age, prolonged major surgery, the use of Patient Controlled Analgesia morphine and pain and discomfort from AACG.

There have been similar case reports of AACG that had a delayed diagnosis peri-operatively. Lotery and Frazer in 1995 reported a case of AACG that was masked by GA. Their patient underwent abdominal hysterectomy and developed nausea, vomiting and an injected red eye on POD 4. Two days after, she became confused and aggressive. She was complaining of eye discomfort, had reduced vision and red eyes. She was diagnosed with AACG on day 11 post-operatively by an ophthalmologist [8]. Lentschener et al in 2002 reported a patient that developed right eye visual loss and bilateral frontal headache 6 hours post-thyroidectomy. The eye discomfort was initially attributed to anaesthesia-related corneal injury. 16 hours post-surgery, the patient became very anxious and agitated, and her eye redness and pain had worsened. She was then diagnosed with AACG by the ophthalmologist on the 24^th^ post-operative hour [9]. These case reports further illustrate the diagnostic challenge of AACG post-operatively.

**Fig. 1.**
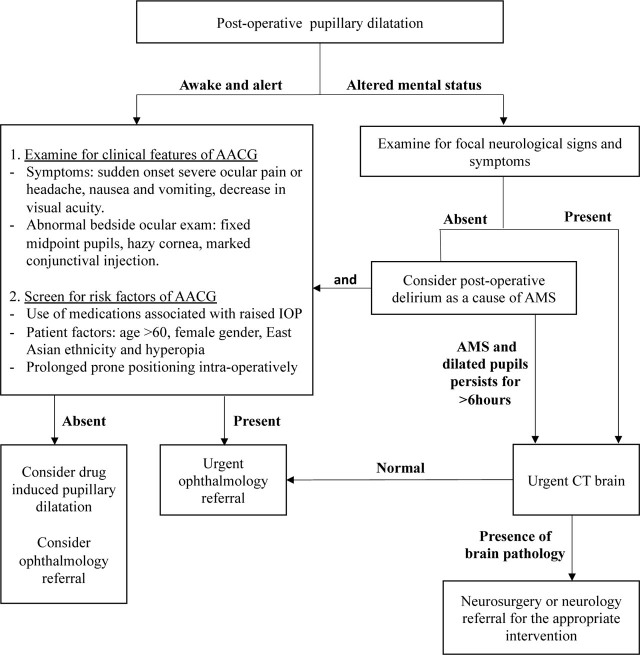
Flow chart to guide the management of post-operative pupillary dilatation.

To the best of our knowledge, AACG and significant altered mental status presenting in an early post-operative period has not been reported before. Our patient was unable to express the typical symptoms of AACG due to her concurrent altered mental status from postoperative delirium. This case demonstrates that the combination of AACG with post-operative delirium could present with signs and symptoms similar to that of a catastrophic brain lesion. In such a scenario, the medical team had to first consider the life-threatening differential of intracranial space occupying lesion. Our patient's airway was promptly secured to provide adequate mechanical ventilation, avoiding secondary brain injury due to hypoxia or hypercarbia. The fluctuations in her GCS and absence of intracranial or 3^rd^ nerve lesions in brain imaging revealed the possibility of dual pathology for her altered mental status with unilateral dilated pupil. The triggers for her AACG attack were likely multifactorial. Her symptoms suggestive of AACG extended beyond the duration of action of medications. Hence, the attack was unlikely to be self-limited and thus, required active treatment to lower her intraocular pressure. Figure 1 is a flow chart to guide the management of post-operative pupillary dilatation.

## Conclusion

In conclusion, AACG needs to be consistently thought of as one of the top differentials in any post-operative patient with eye discomfort or abnormal ocular signs on examination. A referral to the ophthalmologist should be made promptly once AACG is suspected.
